# The Moon-Forming Impact and the Autotrophic Origin of Life

**DOI:** 10.1002/cplu.202300270

**Published:** 2023-10-09

**Authors:** Natalia Mrnjavac, Jessica L. E. Wimmer, Max Brabender, Loraine Schwander, William F. Martin

**Affiliations:** a Department of Biology Institute for Molecular Evolution Heinrich Heine University Duesseldorf Universitaetsstr. 1, 40225 Düsseldorf (Germany)

**Keywords:** acetyl-CoA pathway, carbon dioxide fixation, molecular evolution, moon-forming impact, origin of life

## Abstract

The Moon-forming impact vaporized part of Earth’s mantle, and turned the rest into a magma ocean, from which carbon dioxide degassed into the atmosphere, where it stayed until water rained out to form the oceans. The rain dissolved CO_2_ and made it available to react with transition metal catalysts in the Earth’s crust so as to ultimately generate the organic compounds that form the backbone of microbial metabolism. The Moon-forming impact was key in building a planet with the capacity to generate life in that it converted carbon on Earth into a homogeneous and accessible substrate for organic synthesis. Today all ecosystems, without exception, depend upon primary producers, organisms that fix CO_2_. According to theories of autotrophic origin, it has always been that way, because autotrophic theories posit that the first forms of life generated all the molecules needed to build a cell from CO_2_, forging a direct line of continuity between Earth’s initial CO_2_-rich atmosphere and the first microorganisms. By modern accounts these were chemolithoautotrophic archaea and bacteria that initially colonized the crust and still inhabit that environment today.

## Graphical abstract

**Figure:**
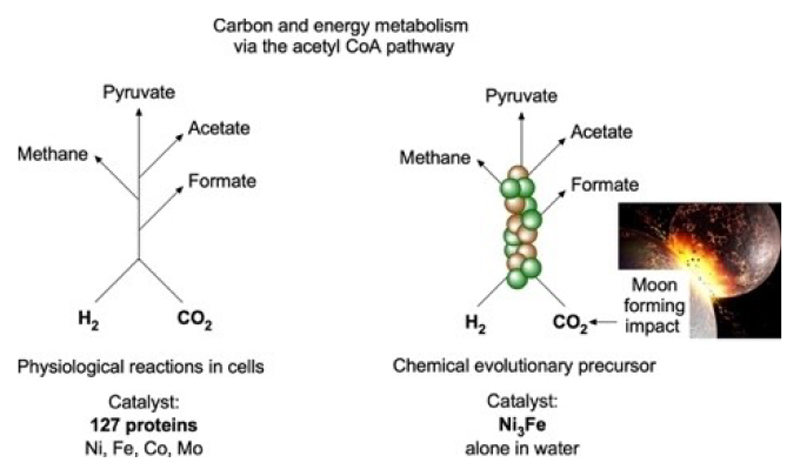
All ecosystems start with autotrophs, cells that convert CO2 to organics. In autotrophic theories for origins, life started from CO2. The Moon-forming impact supplied pure CO2 as the primordial biogenic substrate. Transition metals readily convert H2 and CO2 into formate, acetate and pyruvate, doing the job of 127 enzymes in modern cells, suggesting a facile onset of metabolic origins.

## 1 Formation of the Earth-Moon system, early atmospheres and the origin of life

### 1.1 On the early Earth and the Moon-forming impact

Thoughts on the origin of life are tightly linked to inferences about the chemical environment of the early Earth in a young solar system. The modern Earth is a rocky planet in the habitable zone of the solar system with surface liquid water and an oxidizing atmosphere composed predominantly of N_2_. It harbors tectonic activity and a magnetic field, both consequences of geophysical processes in the Earth’s interior.^[1–2]^ But what about the early Earth?

**Figure 1:**
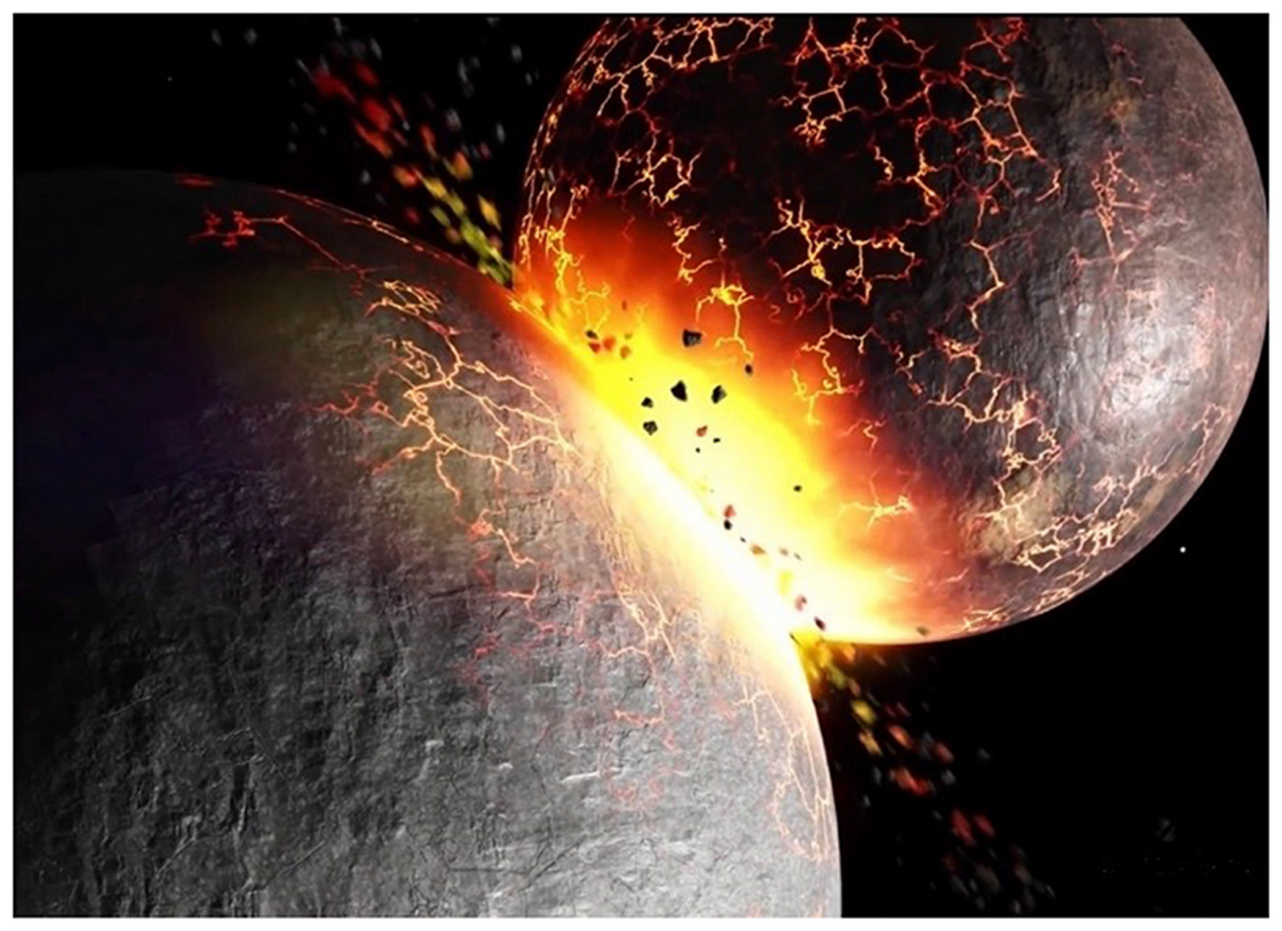
The Moon-forming impact. An artist’s impression of the collision of Theia with Earth. Credit: STEP-ANI-MOTION Studio für Computertrick GmbH, Cologne, Germany.

In the standard model, the Earth formed by accretion of smaller bodies and planetesimals over 4.5 billion years ago (Ga), during a relatively short period of time after the formation of the solar system, accompanied by differentiation: the partitioning of siderophile (iron-loving) elements into the core and lithophile (rock-loving) elements into the silicate mantle.^[3–4]^ The most widely accepted hypothesis for Moon formation is the giant impact hypothesis (Figure 1), which posits that the Moon formed by the collision of a giant Mars-sized impactor named *Theia* with the proto-Earth.^[5–7]^ This occurred shortly after the formation of the solar system, the impact event dating to roughly 4.50 Ga^[8]^ or perhaps as late as 4.35 Ga according to newer findings.^[9]^ The energy released at impact melted the Earth’s mantle into a magma ocean. An estimated 20% of the mantle was vaporized.^[8]^ The atmosphere of rock vapor formed this way was accompanied by degassing volatiles (some N_2_ but mostly H_2_O and CO_2_) that were poorly soluble in the magma ocean, which underwent differentiation.^[10–12]^ The degassing continued as the mantle cooled and solidified over 2–10 million years, giving rise to a secondary steam atmosphere.^[8,11]^

Isotopic evidence indicates that the Earth’s silicate mantle was oxidized, such that its degassing resulted in a secondary atmosphere that was oxidizing, not reducing.^[13–14]^ This is in agreement with evidence from Hadean oxygen fugacities from zircon crystals.^[15]^ Highly reduced gases such as CH_4_ and NH_3_ could have been present transiently, at best, and with short lifetimes, dissociating due to photolysis.^[12–13,16–17]^ Some models suggest the existence of a series of impactors that followed the Moon-forming impact in order to explain the higher-than-expected concentrations of siderophile elements in the Earth’s mantle (the late veneer) and the concentration of Earth’s volatiles as well.^[18–19]^ Other models entail only one additional impactor after Moon formation.^[20–21]^ In an origins context, the main role of these additional impactors is to chemically alter the atmosphere, making it transiently more reducing.^[21–22]^ These additional impactors need to have specific sizes and specific compositions (different types of chondrites have been suggested) if they are to generate the observed veneer and atmospheric NH_3_ and CH_4_.^[21,23–24]^ Alternatively, it has also been suggested that the source of the excess siderophile elements, whose existence gave rise to the idea of a late veneer in the first place, could have simply been the core of Theia – the Moon-forming impactor itself^[25]^ – such that no additional impactors other than Theia are needed to explain the observed mantle composition of some siderophile elements.^[26]^ In that case, there was no late veneer. Recent results by Grewal et al. indicate that the Moon-forming impactor could, on its own, account for the abundance of volatiles on Earth as well.^[27]^ It is thus possible that the series of impactors corresponding to the late veneer never took place, as the evidence for their inferred existence can be explained by the Moon-forming impact itself.^[24–25,27]^

### 1.2 On the secondary atmosphere and the origin of life

The vast majority of water on Earth is thought to predate the Moon-forming impact, stemming from the accretion period.^[8,28–29]^ Water that degassed from the Moon-forming impact generated an atmosphere rich with water vapor. As the Earth cooled, the water vapor condensed and rained to form the oceans. Isotopic evidence from detrital zircons date liquid water on Earth to 4.4–4.3 Ga.^[30–31]^ This left behind a ~100 bar CO_2_-rich atmosphere and a surface temperature of about 500 K.^[11]^ The model by Sossi et al., in which an atmosphere equilibrated with the magma ocean was allowed to cool, indicates that the Earth’s atmosphere following cooling was very similar to that of Venus: mostly CO_2_ (~80 bars) and some N_2_ (~2 bars).^[17]^ Such high CO_2_ concentrations are expected to mitigate the faint young Sun problem and help maintain liquid water on Earth’s surface through a greenhouse effect.^[11,16,32]^

Eventually the CO_2_ in the Earth’s atmosphere dissolved in oceans of liquid water, rendering them slightly acidic. Dissolved CO_2_ then precipitated as carbonates, which were sequestered in the mantle by subduction. Most of the atmospheric CO_2_ was likely subducted by 3.8 Ga.^[11]^ After that the atmosphere was mostly N_2_, like today, but lacking the O_2_ component, which did not come about until the origin of cyanobacterial photosynthesis some 2 billion years later. To summarize, as Zahnle et al. put it *“geological evidence suggests that Earth’s mantle has always been relatively oxidized and its emissions dominated by CO_2_, H_2_O, and N_2_*”^[22]^ or as Sossi et al. put it, Earth likely had *“a prebiotic terrestrial atmosphere composed of CO_2_-N_2_, in proportions and at pressures akin to those observed on Venus*.”^[17]^

How is this relevant for the origin of life? As a consequence of the Moon-forming impact, much, most, or virtually all of the carbon on the proto-Earth was converted to CO_2_ and degassed into the atmosphere, likely going through a short-lived intermediate high temperature phase containing large amounts of CO.^[17]^ The Moon-forming impact converted Earth’s carbon into CO_2_, which is a pure, clean, homogeneous gas that is available to react in the presence of suitable catalysts, providing an excellent starting point for organic synthesis. Today, CO_2_ serves as the entry point of carbon into the carbon cycle, it is the starting point of primary production in all of life’s ecosystems. Under autotrophic theories for the origin of metabolism, the same was true at life’s onset as well. CO_2_ is of course inert by itself, but it is readily converted into organic compounds, provided that a sufficiently strong reductant (H_2_) and suitable catalysts (transition metals) are present. It is the simplest carbon source for life, and the only carbon source required for autotrophic life.

## 2 Autotrophic origins, starting from CO_2_

Life (cell mass) is ~50% carbon by dry weight. It is therefore not surprising that theories for life’s origins are always tied to sources of carbon on the early Earth. At the most basic level, there are two main schools of thought about origins that differ with respect to the source of carbon and energy used by the first cells. In the literature, this dichotomy is sometimes cast in the terms of autotrophic vs. heterotrophic origins, although a division into metabolism-first vs. genetics-first theories generates roughly the same divide. Metabolism-first theories have been around in various guise for a long time. An early paper that is explicit on the issue, is Eakin^[33]^ from 1963: *“hypotheses in which metabolism antedates enzymes and nucleoproteins have been set forth in broad generalities by several scientists including Anker, Bernal, Calvin, Gaffron, Oparin, and Pirie.”* Eakin was thus also not the first, but he does succinctly express the idea that surfaces predate cofactors, which in turn predate proteins: “… *these ancestral cofactors could and did function catalytically without proteins (just as it is possible to so demonstrate with most modern cofactors), and that these primitive organic cofactors (along with inorganic ions) acting on surfaces were the original “bio” catalysts, active long before specific polypeptides evolved*.”^[33]^

### 2.1 Chemolithoautotrophic origins with H_2_ as electron source

Autotrophic theories are older than their heterotrophic counterparts. They are also older than metabolism-first theories. They start with Konstantin Mereschkowsky (1910), who like all of his contemporaries knew next to nothing about carbon metabolism (for lack of its discovery), yet still inferred that life arose when the Earth’s surface was covered with boiling water, and that the first life forms had to be anaerobic thermophiles capable of synthesizing organics from inorganic compounds without photosynthesis (*“Fähigkeit, Eiweiße und Kohlenhydrate, letzteres ohne Vermittlung des Chlorophylls, aus unorganischen Stoffen zu bilden”*).^[34]^ In modern terms, that translates to a chemolithoautotrophic origin of life, even though in 1910 no one knew how cells fix CO_2_. After the discovery of the Calvin cycle,^[35]^ the reductive TCA cycle^[36]^ and the acetyl-CoA pathway,^[37–38]^ Georg Fuchs and his team worked out half of the known pathways of CO_2_ fixation.^[39]^ The distribution of the acetyl-CoA pathway in distant anaerobic lineages of both prokaryotic domains, its low energy requirements and its versatility for the assimilation of various one-carbon and two-carbon compounds prompted Fuchs and Stupperich to propose that the acetyl-CoA pathway of carbon fixation is the most ancient among the CO_2_ fixation pathways known at the time.^[40]^ This meshed well with the presence of the pathway in anaerobic autotrophs that lack cytochromes and that had previously been suggested to be primitive, notably clostridial acetogens^[41]^ and methanogens.^[42]^ It also meshed well with Wächtershäuser’s later proposals for autotrophic origins,^[43]^ although it should be mentioned that Wächtershäuser used CO as a starting material in his experiments, rather than CO_2_.^[44–46]^ The catalysts he used to test the theory of an iron-sulfur world^[47]^ were Fe and Ni sulfides, which perform one electron reactions. Wächtershäuser argued that the electrons for the first organic syntheses stemmed from the formation of pyrite (FeS_2_) and opposed the view that H_2_ was the original reductant for CO_2_′^[43]^ because the redox potential of H_2_ at pH 7 and 1 atm H_2_ (*E*_0_^′^ = −414 mV) is not sufficiently negative to reduce CO_2_.^[48]^

However, recent studies show that H_2_ can readily reduce CO_2_ to several intermediates and end-products of the acetyl-CoA pathway using only transition metal catalysts in the laboratory under the conditions of serpentinizing hydrothermal vents, which are realistic environments for prebiotic chemistry and the origin of life under autotrophic theories.^[49–51]^ A curious question arises from those observations – how, from an energetic standpoint, can H_2_-dependent reduction of CO_2_ be facile in the laboratory if the midpoint potential of H_2_ under standard conditions is insufficient?

The answer is that i) serpentinizing systems do not harbour standard physiological conditions (pH 7 and 1 atm of H_2_) and ii) that laboratory simulations of CO_2_ fixation under simulated hydrothermal vent conditions are typically performed under alkaline conditions in order to simulate the effluents of serpentinizing systems, which present a pH in the range of 9–11 or higher.^[52–56]^ Because the effluent of serpentinizing systems is alkaline and H_2_ rich, often 10 mM H_2_ and more,^[57]^ and the redox potential of the 2H^+^/H_2_ pair is pH-dependent, the redox potential in serpentinizing systems (and laboratory simulations thereof) is on the order of −700 to −800 mV (Table 1).^[57–59]^ This supplies the reducing power necessary for CO_2_ reduction to proceed, provided that suitable metal or mineral surfaces such as Ni_3_Fe as catalysts are present.^[49]^ The first reduced carbon compounds relevant for the origin of life could have formed from H_2_ and CO_2_. Serpentinizing systems have immense reducing power within the range of biologically relevant reactions. They can convert CO_2_ to organics, N_2_ to NH_3_, possibly phosphate to phosphite (recently reviewed by Schwander et al.^[58]^) and might have permitted the reduction of FeS clusters of ferredoxin (*E^′^* of ca. −500 mV under cytosolic conditions), before the evolution of hydrogenases.^[48]^

Another observation favours H_2_ as the ancestral reductant. Modern H_2_-dependent chemolithoautotrophs such as acetogenic bacteria and methanogenic archaea readily reduce CO_2_ with electrons from H_2_. This is fully in line with theories for autotrophic origins,^[34,39,43]^ but H_2_-dependent chemoautotrophs have to go to great lengths using enzymes that perform flavin-based electron bifurcation in order to generate reduced ferredoxin for CO_2_ reduction.^[48]^

### 2.2 The importance of flavin-based electron bifurcation for early metabolism

Flavin-based electron bifurcation is a mechanism that enables cells to reduce low potential ferredoxin with electrons from H_2_ at pH 7 by a flavin that splits (bifurcates) the electron pair from hydrogen across two acceptors: one with higher potential and another one with lower potential. The reduction of the higher potential acceptor is energetically favorable and is coupled to the endergonic reduction of a low potential acceptor which, in the case of CO_2_ fixation, is ferredoxin. Though only discovered in 2008,^[60]^ flavin-based electron bifurcation is essential and apparently universal in the physiology of strictly anaerobic prokaryotes.^[61–62]^ The mechanisms of flavin-based electron bifurcation have been studied in some detail.^[63–64]^

Although not required for nonenzymatic CO_2_ reduction by H_2_ in alkaline conditions, electron bifurcation allows cells, both modern and ancient ones, to exploit the reductive potential of environmental H_2_ at pH 6~7 even at low H_2_ partial pressures near 10^−5^atm^[65]^ (compare Table 1), forging a link between metabolism and environment.^[48]^ The issue of ferredoxin reduction with electrons from H_2_ intuitively leads to thoughts about early evolution. Lipmann (1965) wrote *“I find it possibly of relevance that hydrogen activation […] is mediated by one of the more primitive catalysts, the recently discovered ferredoxin.”*^[41]^ In a similar vein, Eck and Dayhoff (1966) wrote of ferredoxin (which we today know to be an electron carrier, not a catalyst) *“It catalyzes the synthesis of pyruvate from carbon dioxide and acetylcoenzyme-A. This indicates its involvement with one of the simplest, most primitive synthetic processes in intermediary metabolism, the fixation of CO_2_. It participates in nitrogen fixation and hydrogenase-linked reactions*.”^[66]^ The findings that ferredoxin and other proteins with FeS clusters were, in essence, using rocks as their prosthetic group^[67]^ clearly suggested their antiquity.

### 2.3 Origins in serpentinizing hydrothermal systems

The chemistry of submarine hydrothermal fields^[68]^ aligned well with ideas about early physiological evolution and quickly led to explicit proposals for an origin of life at deep sea hydrothermal vents.^[69–70]^ Theories of H_2_-dependent, chemolithoautotrophic origins converge effortlessly with the chemistry of serpentinizing deep sea hydrothermal systems,^[71–72]^ where geochemical reactions driven by and catalyzed by transition metals produce large amounts of H_2_ and abiotic formate, the first intermediate of CO_2_ fixation via the acetyl-CoA pathway,^[52]^ as well as methane, the end product of methanogenesis via the acetyl-CoA pathway.^[73]^ In serpentinization-dependent autotrophic theories, the main carbon converting geochemical reactions are homologous – similar by virtue of common ancestry – to biochemical reactions in the acetyl-CoA pathway.^[74]^ The underlying premise is that the environment of the early Earth can give rise to biochemicals via geochemical reactions with the help of transition metal catalysts that resemble those in metabolism. This physiological constraint naturally generates a chemically continuous transition from non-life to life.

That brings us back to the Moon-forming impact and the source of carbon for the origin of metabolism, CO_2_, and autotrophic theories. The Moon-forming impact gave rise to a pure and indefinitely stable, inert form of carbon in the gas phase, CO_2_. In order to give rise to metabolism, CO_2_ required activation on the surface of transition metal catalysts and reduction by H_2_. In the laboratory, this generates formate, acetate and pyruvate, the backbone of microbial carbon and energy metabolism overnight.^[49]^ Extension of the C3 carbon backbone by further ferredoxin-dependent CO_2_ incorporations, in addition to the metal-catalyzed steps in the reverse TCA cycle,^[75–77]^ or via an aldol condensation akin to the one in the gluconeogenic pathway,^[78–80]^ generates the carbon backbones for amino acid biosynthesis. In metabolism, nitrogen is incorporated as NH_3_ through reductive amination or transamination of 2-oxoacids at the final steps of amino acid biosynthesis, early metabolic evolution likely followed a similar path.^[43,74,80–81]^

In many enzymatic biosynthetic reactions, C–N bond formation involves nucleophilic attack of a carbonyl carbon by an amino group nitrogen, and often requires activation through phosphorylation by ATP. Phosphorylation aids the reaction in several ways, inter alia by increasing the electrophilicity of the carbonyl carbon and by generating a good leaving group that acts as a dehydrating agent.^[82]^ However, recent studies show that C–N bond formation can take place in the absence of a phosphoryl donor under hydrothermal conditions using transition metal catalysts.^[83]^ In that study, Ni–Fe nitrides were synthesized under ammonia flow at deep crust temperatures (300 to 400°C). The inorganic catalysts contained N activated as nitrides, and generated formamide (and acetamide in some cases) over a range of temperatures and pH, starting from CO_2_ and either water or H_2_ as the electron source. The point here is that under geochemical conditions Fe–Ni catalysts can forge C–N bonds without the participation of phosphate. In addition, recent experiments have shown that serpentinization can be a stable source of ammonia,^[84]^ which is generated during the process from N_2_ (present in the primordial atmosphere)^[17]^ and H_2_ (produced through serpentinization). The presence of CO_2_ accelerates ammonia formation.^[84]^

### 2.4 Energetics of prebiotic reactions in serpentinizing hydrothermal systems

The central pillar of autotrophic theories is that the reactions of primitive non-enzymatic microbial metabolism, starting from CO_2_, had enough specificity and sufficient flux rates to support the origin of non-enzymatic (metal-catalyzed) protometabolic networks leading to amino acids, nucleobases and cofactors as building blocks and catalysts for further chemical evolution.^[85–86]^ Most of the biosynthetic reactions of core metabolism are exergonic under the conditions of serpentinizing hydrothermal vents,^[59]^ although some remain endergonic and involve the participation of a phosphoryl donor. Potential prebiotic energy currencies have been widely discussed in the literature.^[74,87–91]^ Acyl phosphates stand out as obvious candidates because of their ability to phosphorylate ADP due to their higher phosphorylating potential.^[92–93]^ In addition, acetyl phosphate is a much simpler molecule than ATP, and is synthesized during acetogenesis via the acetyl-CoA pathway.^[74,88]^ Non-enzymatic acetyl phosphate formation from thioacetate has been reported.^[94]^ Thioesters have also been considered as prebiotic energy currencies, not least because they are found as intermediates in reactions leading to substrate-level phosphorylation.^[74,95–96]^ Recent studies suggest the possibility of abiotic thioester synthesis in Hadean deep-sea vent environments.^[97]^ Another form of phosphorus, phosphite, was recently reported in serpentinite rocks, suggesting it can form during serpentinization.^[98]^ It has been known for a while that some bacteria are capable of oxidizing phosphite to phosphate, which has been suggested to be an ancient trait.^[99]^ Buckel proposed an ancient mechanism of substrate-level phosphorylation of ADP by phosphite via an acylphosphite and an acylphosphate intermediate.^[100]^ In addition, phosphite is more soluble than phosphate, presenting a possible solution to the widely discussed ‘phosphate problem’ at the origin of life.^[101]^ Recent metagenomic studies point to an enrichment in phosphonate and phosphite metabolizing and transporting proteins in microbial communities of reducing hydrothermal systems,^[102]^ suggesting a potential role for phosphite in modern serpentinizing systems and possibly, by inference, at the origin of metabolism.

Some authors argue in favor of wet-dry cycles to circumvent the need for phosphoryl donors in order to make the first dehydration/condensation reactions energetically downhill.^[103–105]^ Wet and dry polymerization is often thought to involve surfaces onto which molecules adsorb^[106]^ increasing local concentrations or enhancing catalytic properties. For example, biological molecules can bind and chelate surface transition metal minerals or silica via carboxylate groups,^[107–108]^ with multiple effects on the reaction parameters, such as making carbonyls more electrophilic, a function typically carried out by phosphorylation.^[108]^

There is a common misconception that deep-sea vent environments are necessarily high in water activity. Water is actually consumed by very dry rock during the serpentinization process, which can also lead to a local increase in salinity (ionic strength) and a decrease in water activity.^[109]^ Serpentinization slows down and eventually comes to a halt at very low water activities, such that seawater has to diffuse from the outside into the rock pores through newly formed cracks in the crust in order for the process to continue – a scenario reminiscent of wet-dry cycles.^[109–110]^

Autotrophic theories currently posit that the central reactants of core microbial metabolism tend to unfold from CO_2_, H_2_ and NH_3_ as a set of thermodynamically metastable intermediates in the presence of suitable inorganic catalysts. Given recent advances in laboratory CO_2_ fixation with H_2_,^[49–51,111]^ in amino acid synthesis with transition metals,^[75–76,112–113]^ in synthesis of nucleotide constituents^[114–115]^ and in metal-dependent redox reactions involving cofactors,^[116]^ such a proposition seems less radical now than it did 20 years ago. Abiogenic synthesis of amino acids has been reported from hydrothermal systems,^[53,117]^ but abiogenic nucleic acid components have not. Nonetheless, congruence and overlap between reactions of serpentinizing systems connect the metabolism of primitive microbes to the chemistry of the early Earth.

## 3 Heterotrophic origins, starting from more reduced carbon sources

Theories for heterotrophic origins generally take root in the concept of organic soup presented by Oparin and Haldane in the 1920s.^[118–119]^ The experiment by Miller and Urey that generated amino acids and other organic compounds from methane, ammonia and water under electric discharge provided a means of synthesizing organic soup.^[120–121]^ Oró’s synthesis of adenine from ammonium cyanide^[122]^ provided a simple chemistry to nucleobases. Generations of chemists used similar conditions in laboratory experiments to synthesize the building blocks of life. In many cases these experiments require reduced starting compounds that could only be provided under a reducing early atmosphere.^[21]^ Sometimes the reaction steps require *uv* radiation, which then ties the entire chemistry to a terrestrial surface,^[123]^ in other cases the location for prebiotic chemistry is not strictly defined.^[124]^ The synthesis of nucleo-bases from cyanide condensations^[122]^ and nitriles^[123,125]^ naturally joined with the concept of an RNA world, which had emerged with the discovery of catalytic RNAs in the early 1980s^[126–127]^ and aligned well with Spiegelman’s and Eigen’s experimental and theoretical work from the late 1960s to the 1980s on *in vitro* selection among replicating RNA molecules.^[128–132]^ With RNA demonstrably able to fulfill the informational function of DNA and some catalytic tasks usually attributed to proteins, the ability to synthesize nucleotides and RNA was regarded by many as key to solving the origin of life problem. In an RNA world, the circumstances surrounding the origin of enzymatic metabolism are of secondary importance. Recent studies highlight the potential for very interesting and informationally relevant chemistry at the interface of the RNA and the peptide world,^[133]^ calling strict dichotomies between RNA and peptide evolution into question.

## 4 Autotrophic, heterotrophic, pros and cons

Divisions in schools of thought about origins are still evident.^[134]^ In the same way that metabolism-first (autotrophic origin) theories fall short (so far) on the laboratory synthesis of nucleobases from CO_2_ and NH_3_ and therefore lack a mechanistic connection to replication, a shortcoming of the genetics-first view is that crucial ingredients used for the synthesis of nucleobases – nitrile moieties – do not occur in reactants or products of core microbial metabolism. Cyanide and nitriles are very efficient in the laboratory synthesis of bases, as is formamide,^[135]^ but neither formamide nor nitriles occur in the biosynthetic routes used by cells, leaving no options to directly connect modern core biochemistry in an evolutionary inference to an origins scenario that starts from cyanide, nitriles or formamide.

### 4.1 The origin of reduced carbon compounds in a postimpact atmosphere

In favor of autotrophic origins, CO_2_ meshes well with modern life and with primordial atmosphere. CO_2_ directly interfaces with metabolism at over 400 reactions.^[136]^ Life on Earth ultimately synthesizes all of its components from CO_2_ and the most ancient pathway of CO_2_ fixation entails a chemistry that merges seamlessly with that of serpentinizing hydrothermal systems. The requirement for CO_2_ is in full agreement with current views regarding the composition of the atmosphere after the Moon-forming impact.^[13–14,17]^ Organic syntheses from CO_2_ necessarily require a reductant – H_2_, and a nitrogen source – NH_3_, for the generation of nitrogenous compounds. Both are continuously synthesized within the crust by serpentinization.^[137–138]^ H_2_ is the same reductant that the acetyl-CoA pathway uses and it was the source of electrons for primary production prior to the origin of photosynthesis. Conveniently, serpentinization provides H_2_ exactly where it is needed for origins, at hydrothermal vents^[74]^ such that H_2_ and CO_2_ interface in an environment where the same minerals that catalyze CO_2_ reduction are formed.^[138]^

In favor of cyanide, nitriles and formamide, they reliably and reproducibly enable the synthesis of nucleobases in the laboratory. If base synthesis is the main criterion for a prebiotic chemical scenario, cyanide and nitriles (and formamide) are the starting materials of choice. But cyanide and nitriles are not among the atmospheric constituents following the Moon-forming impact in current models.^[13–15,27]^ In addition, volcanic plumes do not appear to be significant cyanide sources.^[139]^ The RNA world in its current formulation struggles somewhat with the availability of the necessary precursors for its proposed organic syntheses on the early Earth. Because the early atmosphere was not a reducing atmosphere as Miller and Urey believed,^[121]^ and because the necessary nitrile precursors could not form in an oxidizing atmosphere from CO_2_, proponents of heterotrophic origins have suggested that additional impactors following the Moon-forming impact could have transiently transformed the atmosphere from an oxidizing state (H_2_O, CO_2_, N_2_) to a short-lived reducing state (NH_3_, CH_4_, H_2_). This would create an environment that could support origin of life scenarios that require reducing atmospheric conditions^[21–22]^ and reduced precursors such as hydrogen cyanide and other nitriles in contact with sunlight.^[21]^ In extreme formulations, this gives rise to immense and concentrated, but inferred, ’stockpiles’ of cyanide on the early Earth.^[140]^

### 4.2 What does LUCA say?

Life is a set of (bio)chemical reactions, the oldest of which date at least to the last universal common ancestor (LUCA). In hydrothermal versions of autotrophic origins, no major shifts in the basic chemical reactions of life are required in the transition from origins to LUCA and later to the first free-living cells: The first CO_2_-reducing reactions set the pattern of products in the acetyl-CoA pathway, LUCA lived from gasses in a hydrothermal environment and made extensive use of both transition metals and cofactors, and the first free-living cells were acetogens and methanogens, which use the acetyl-CoA pathway and obtain both their carbon and energy from the reduction of CO_2_ with H_2_.^[74,96,141–142]^ In this very explicit metabolism-first model, the exergonic reactions fueling the first organic syntheses and the first free-living cells remained constant while the nature of the catalysts changed as evolution progressed.^[142]^ In genetics-first models, the connections between the first organic-synthetic reactions leading to RNA and the energetics of LUCA and the first cells are not readily specified, in part because genetics-first models account in great detail for replication and selection,^[143–145]^ but not for carbon and energy metabolism that underpin the genetic process. The origin of nucleoside phosphates are one thing, the origin of cells, and of life are another.^[134]^ Just as metabolism-first theories still fall short on the origin of genetic coding, genetics-first approaches have yet to naturally dock into the reactions of microbial metabolism.

### 4.3 Molecular fossils

The antiquity of RNA catalysis is indisputable – the ribosome itself is a ribozyme^[146]^ and the evolution of all modern proteins postdates the origin of the ribosome and the genetic code.^[147]^ However, at present only 21 catalytic RNA molecules are known across all life, according to the Ribocentre database.^[148]^ Even though the catalytic efficiency of ribozymes can be shown to be comparable to protein enzymes for some reactions,^[149–151]^ the types of reactions catalyzed by ribozymes in nature are limited: peptidyl-transfer in the ribosome, transesterifications or phosphate hydrolysis reactions.^[152–153]^ Part of the popular appeal of genetics-first models resides in the application of Darwinian evolution to RNA, invoking natural variation and natural selection among molecules before the origin of cells. In that sense, genetics-first presents a universally tangible evolutionary mechanism – Darwinian evolution – that remains constant across the divide that connects the first organic synthesis to the first replicating cells. Many catalytic RNAs with demonstrable RNA polymerizing activities have been developed in the laboratory,^[143,145,154]^ although the corresponding activities have not been identified in natural cells. If ribosomal RNA arose from replicating RNA, one might ask: where is its complementary strand? Curiously, the first hints for strand complementarity in molecular evolution trace to a protein-coding gene, in which the two complementary strands of the same DNA encode the ancestral forms – called “urzymes” by Carter, Wolfenden and colleagues – of the two classes of aminoacyl-tRNA synthetases.^[155–157]^ In a world where RNA mainly synthesized protein, DNA might be more ancient than most of us currently think.^[158]^

While in the heterotrophic origins scenario modern ribozymes are seen as molecular fossils of a time when RNA catalyzed a broad set of reactions before the emergence of protein enzymes, autotrophic origins scenarios imply that the role of RNA, albeit important, was always limited to informational processing. In modern autotrophic theories, early metabolic reactions were catalyzed by transition metals, cofactors, and ultimately proteins, not RNA. Most redox reactions in life require redox-active transition metals (Fe, Ni, Co, Mo, Mn) and/ or redox active organic coenzymes and prosthetic groups such as NAD^+^, FAD, FMN and F_420_, cofactors which are bound by enzymes today. These coenzymes and prosthetic groups likely represent molecular fossils of ancient chemistry catalyzed by transition metals^[49,80,108,113,159–160]^ and by cofactors^[116,161–163]^ in the time before the existence of genetically encoded peptides.

## 5 CO_2_ and primary production underpin all ecosystems

### 5.1 CO_2_ fixation and early metabolism

All carbon in today’s life ultimately stems from CO_2_. In modern ecosystems, primary producers fix CO_2_ by any of the seven carbon fixation pathways known.^[39,164]^ The resulting reduced organic compounds comprise cell mass and metabolic end-products that serve as food for heterotrophs. Life forms exist today in environments with supercritical CO_2_ concentrations, and such high environmental CO_2_ can push metabolism in the direction of CO2 fixation rather than respiration, if the cell has the proper enzymatic machinery.^[165]^

In modern ecosystems, the global rate of photosynthetic primary production (CO_2_ fixation) is estimated to be roughly 10^5^ times greater than that of H_2_-dependent chemolithotrophs that populated the Earth in Hadean-Archaean times.^[166]^ The Hadean-Archean rate of roughly 7×10^11^ g of assimilated inorganic C per year, averaged across the surface of the Earth (5×10^14^ m^2^) corresponds to about 0.0014 g of C or 0.003 g of cells (dry weight) per m^2^ per year or, very roughly, as cell size can vary by more than 2 orders of magnitude depending on growth rate, to about 10^10^ cells per m^2^ per year. That is a substantial amount of autotrophic growth in Hadean times, sufficient to support the origin of heterotrophic lifestyles^[167]^ and later photosynthesis.^[168]^

Primordial primary production had to have been H_2_-dependent because there are no other environmentally available electron donors with sufficiently negative redox potentials to reduce CO_2_ for growth. New forms of H_2_-dependent metabolism are still being discovered in serpentinizing hydrothermal systems.^[53]^ Though native metals such as Fe^0^ can serve as the electron source to support growth of acetogens^[169]^ and methanogens,^[170]^ Fe^0^ reacts with water to generate Fe^2+^ and H_2_ such that both in modern environments and in an origins context, native metals generate H_2_ as the reductant. Thus, from a modern perspective, the first autotrophs were probably chemolithoautotrophs, just as Mereschkowsky suspected,^[34]^ but today we can be more specific with regard to their likely metabolism. They were hydrogenotrophic, most probably employing hydrogenotrophic methanogenesis^[171–172]^ and hydrogenotrophic acetogenesis.^[54,141]^

**Figure 2:**
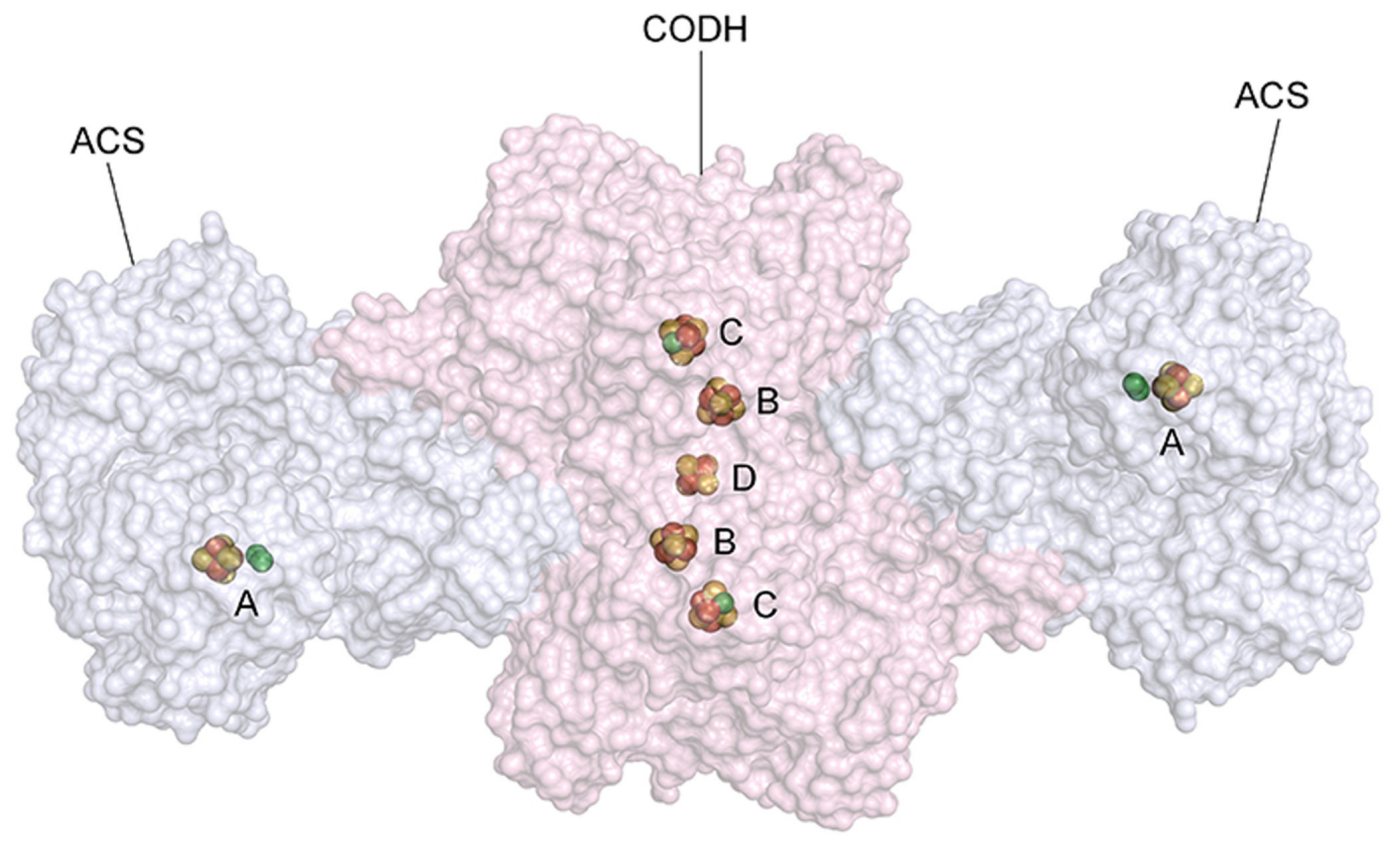
Crystal structure of the conserved bifunctional enzyme carbon monoxide dehydrogenase/acetyl-CoA synthase (CODH/ACS) from the acetyl-CoA pathway (PDB ID: 7ZKJ).^[181]^ The CODH homodimer is surrounded on both sides by an ACS subunit. The metal clusters are labeled, namely the D and B cubane Fe_4_S_4_ clusters of CODH that serve an electron transfer function, the C (NiFe_4_S_4_) cluster of CODH where the reaction occurs, and the active site A cluster of ACS, which is a Fe_4_S_4_-type cluster bridged to a binuclear Ni—Ni site.^[176]^ The figure was prepared with PyMol (The PyMOL Molecular Graphics System, Version 2.5.4, Schrödinger, LLC).

**Figure 3:**
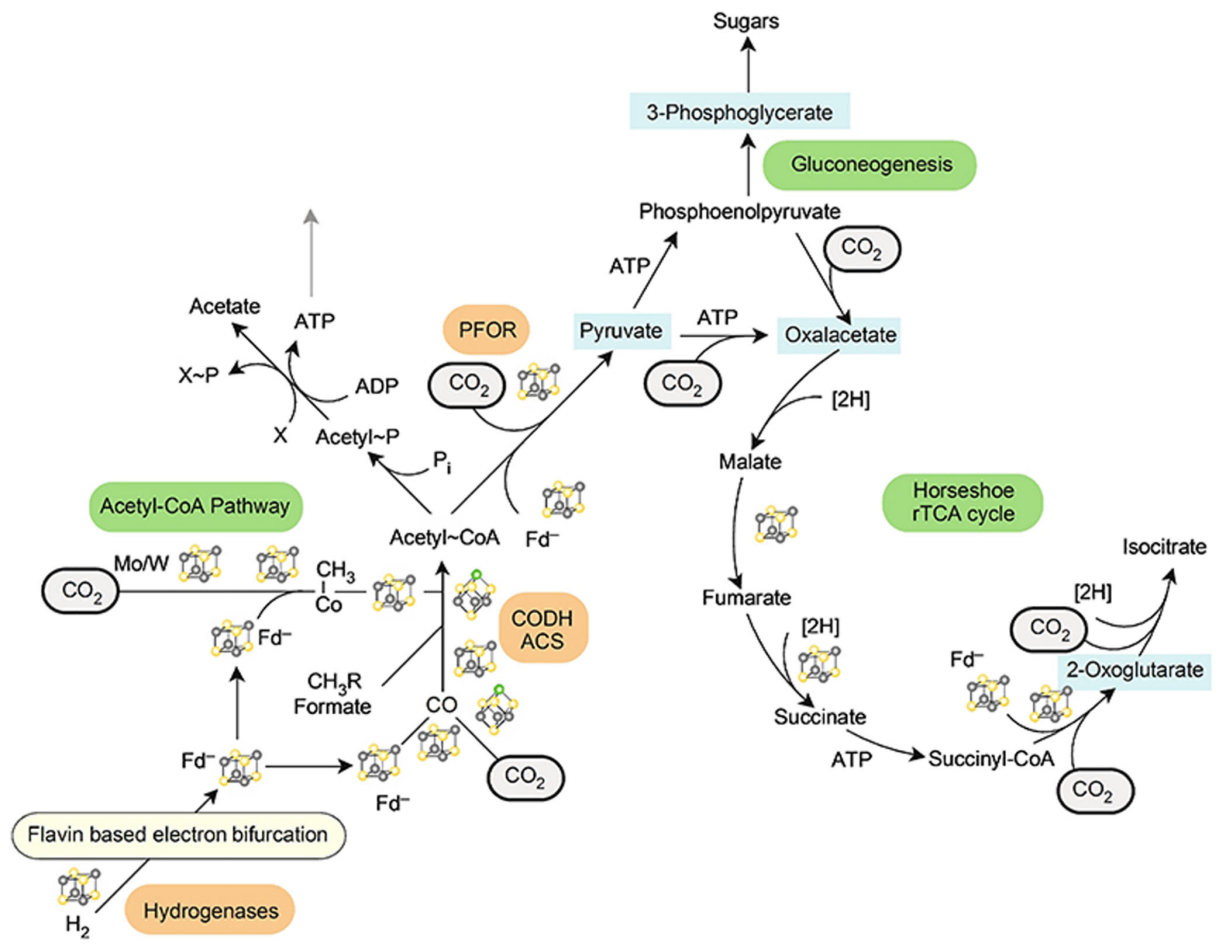
The enzymatic core metabolism of early life forms, modified from^[74,179,182]^. Environmentally produced H_2_ from serpentinization could directly reduce CO_2_ made accessible by the Moon-forming impact in metal-catalyzed geochemical reactions under hydrothermal conditions. The transition to an enzymatically catalyzed protometabolism, shown here, involved a gradual decrease in dependency on vent conditions, whereby H_2_ became the electron donor for ferredoxin reduction catalyzed by hydrogenases, which employ flavin-based electron bifurcation in order to couple the endergonic reduction of ferredoxin with the exergonic reduction of a higher potential acceptor.^[61,63–64]^ CO_2_ is fixed in the acetyl-CoA pathway, with ferredoxin as the electron donor in reactions of both the methyl and the carbonyl branch.^[39]^ While the methyl branch is catalyzed by a series of enzymes that are non-homologous in bacteria and archaea, the carbonyl branch is catalyzed by the conserved bifunctional carbon monoxide dehydrogenase/acetyl-CoA synthase (CODH/ACS).^[136,174,176,183]^ The product of the pathway, acetyl-CoA, is a high-energy thioester that can generate phosphoryl donors such as acetyl-phosphate, which has been proposed to be a primordial energy currency,^[74,94]^ and ATP, via substrate-level phosphorylation. These reactions are catalyzed by phosphotransacetylase and acetate kinase, respectively.^[184–185]^ In acetogens (bacteria) and methanogens (archaea) the reduction of CO_2_ via the acetyl-CoA pathway is furthermore coupled to the generation of an ion gradient and the harnessing of its electrochemical potential for ATP synthesis by an ATP synthase.^[65,186–187]^ The acetyl-CoA produced in the acetyl-CoA pathway is partly used to generate building blocks. Pyruvate:ferredoxin oxidoreductase (PFOR) catalyzes the reductive carboxylation of acetyl-CoA to pyruvate,^[165,177]^ which is then directed into gluconeogenesis^[78,188–189]^ and the incomplete (linear) rTCA pathway,^[178]^ generating central metabolic intermediates for further synthesis of amino acids, cofactors and nucleobases. Six reactions of the rTCA cycle have been shown to proceed non-enzymatically, catalyzed by transition metals, and three of them (the sequence from oxaloacetate to succinate in the linear rTCA) have been obtained with hydrogen as the reductant.^[75,77]^ Other reports of non-enzymatic variants of the reactions in the figure are mentioned in the text and summarized in^[182]^. Biological reductants other than Fd^−^ (reduced ferredoxin) are indicated with [2H]. Participation of FeS or FeNiS clusters in the enzymatic reactions is indicated.

In methanogens and acetogens growing on H_2_, the acetyl-CoA pathway (Wood-Ljungdahl pathway) converts H_2_ and CO_2_ to formate, which is further reduced through a series of C1 compounds bound to pterin cofactors (tetrahydrofolate in acetogens and tetrahydromethanopterin in methanogens) to a methyl group that is transferred to a corrinoid iron-sulfur protein. This is the methyl branch, catalyzed by six enzymes. The carbonyl branch is catalyzed by only one enzyme, albeit an important one – bifunctional carbon monoxide dehydrogenase/acetyl-CoA synthase (CODH/ACS).^[39,173]^ CODH catalyzes the two-electron reduction of CO_2_ to CO on its C-cluster (Ni–Fe–S), with ferredoxin as the electron donor.^[174–175]^ The CO molecule diffuses through a gas channel to the ACS subunit. In the ACS active site, a condensation reaction of CO with the methyl group from the corrinoid iron-sulfur protein occurs on a Ni atom in the ACS A-cluster (Figure 2).^[173,176]^ This generates a Ni-bound acetyl group that is removed from the enzyme by coenzyme A via thiolysis. The resulting acetyl-CoA can be converted to pyruvate as the central compound of metabolism by incorporation of one more CO_2_ via the ferredoxin-dependent enzyme pyruvate:ferredoxin oxidoreductase (PFOR).^[177]^ About 50% of the metabolic flux channeled into biosyntheses stems from acetyl-CoA and pyruvate.^[39]^ Pyruvate synthesis allows for the carbon flux to be channeled into gluconeogenesis for sugars and into the incomplete (linear) reverse TCA, generating oxaloacetate and 2-oxoglutarate as central metabolic intermediates (Figure 3).^[178–179]^ The acetyl-CoA pathway is a versatile metabolic route that can be employed in the assimilation of several simple organic compounds, such as formate, methanol, and formaldehyde.^[39,180]^ In addition, it can release enough energy to allow cells to generate ion gradients without involving high-energy phosphorylated compounds. It is the only energy-releasing CO_2_-fixation pathway, integrating ATP synthesis with CO_2_ fixation, which makes it a likely candidate for the first metabolic pathway on Earth.^[40]^

### 5.2 A geochemical analogue of the acetyl-CoA pathway corroborates its antiquity

**Figure 4:**
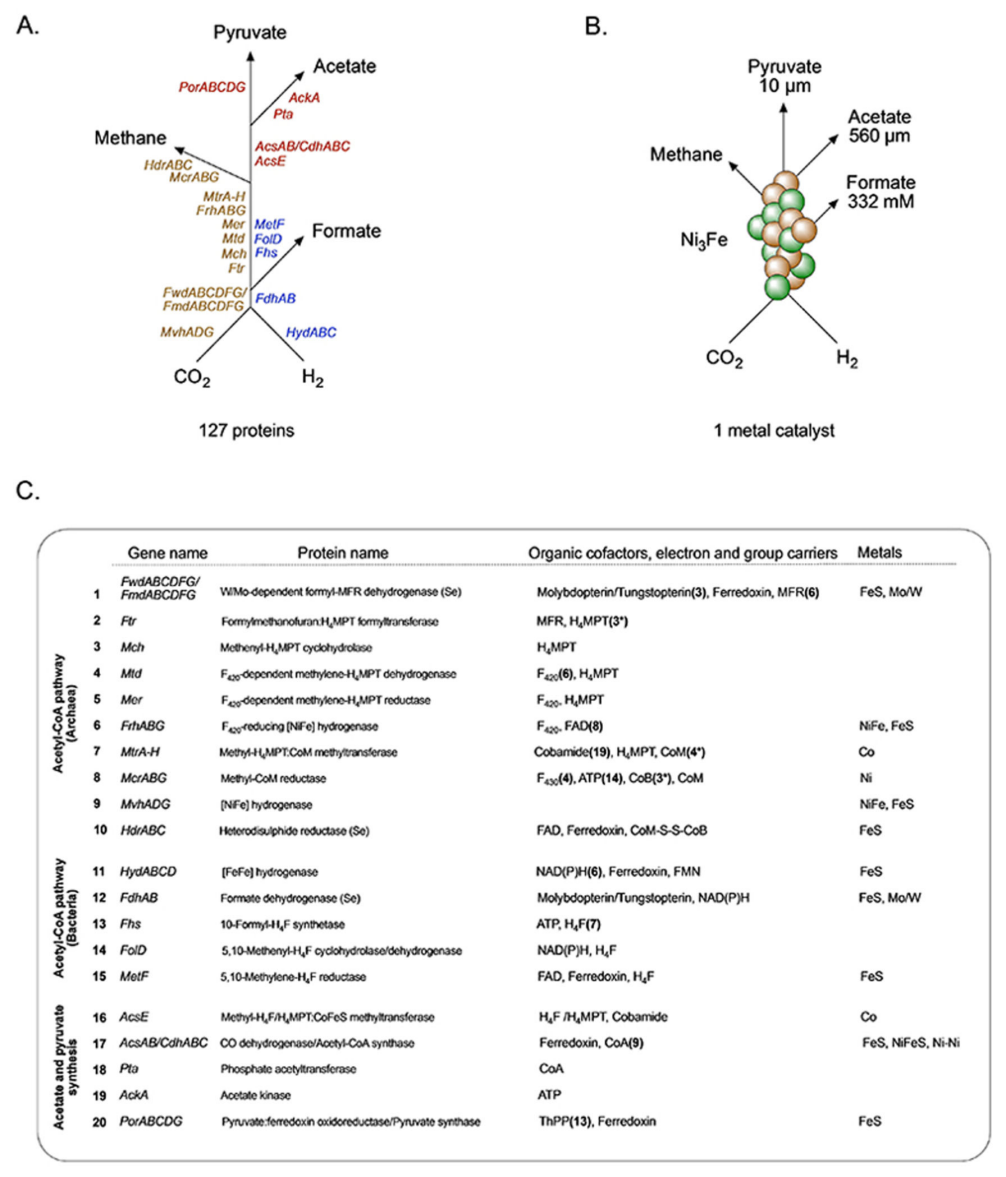
A. The genes coding for the enzymes of the acetyl-CoA pathway are shown in order. Archaeal methanogenic enzymes are in brown, bacterial acetogenic enzymes are in blue, while enzymes present in both domains are shown in red. The latter include the carbonyl branch and the synthesis of acetate and pyruvate. In addition to the 20 enzymes of the pathway itself, 2 carrier proteins are required (ferredoxin and the corrinoid iron-sulfur protein) and at least 105 enzymes to synthesize the cofactors essential to the pathway (several cofactor biosynthesis pathways are not fully characterized). B. Analogues of the acetyl-CoA pathway have been obtained in the laboratory under alkaline hydrothermal vent conditions. Awaruite (Ni_3_Fe) formed in serpentinizing systems catalyzes the formation of methane, formate, acetate and pyruvate, which are intermediates and products of the acetyl-CoA pathway, from CO_2_ and H_2_.^[49,111]^ Reaction yields are shown for a 16 h reaction at 100 °C and pH 8 with 0.625 mmol Ni_3_Fe.^[49]^ Pyruvate has been found to react further to form citramalate,^[50]^ a metabolic intermediate in some organisms (not shown).^[198–199]^ Only one catalyst is required. C. The 20 genes and enzymes of the reductive acetyl-CoA pathway to pyruvate in bacteria and archaea are shown.^[65,176,181,192,200–207]^ Selenoproteins are marked (Se). For each enzyme the cofactors and transition metals that participate in the reaction are listed. The number in brackets next to the first appearance of each cofactor shows the number of enzymes required for its biosynthesis. When the enzyme number is uncertain (likely an underestimate) because the biosynthetic pathway has not been fully characterized, the enzyme number is followed by an asterisk (*). Abbreviations: MFR: methanofuran; H_4_MPT: tetrahydromethanopterin; CoM: coenzyme M; CoB: coenzyme B; H_4_F: tetrahydrofolate; CoFeS: corrinoid iron-sulfur protein; CoA: coenzyme A; ThPP: thiamine pyrophosphate.

The acetyl-CoA pathway from H_2_ and CO_2_ to pyruvate requires 20 enzymes and 14 organic cofactors, coenzymes and C1 carriers (Figure 4). The synthesis of each of these depends on several enzymes (Supplementary Table 1). In total, the synthesis of formate, acetate, pyruvate and methane from H_2_ and CO_2_ in acetogens and methanogens requires 127 proteins at the bare minimum. Kaster et al. have surmised that 200 genes are required for methanogenesis.^[190]^ The enzymes of the acetyl-CoA pathway abound in transition metal clusters,^[39,173–174]^ which need to be assembled and incorporated (Figure 4C). In addition, two protein carriers are indispensable for the pathway – ferredoxin and the corrinoid iron-sulfur protein CoFeS. CoFeS uses cobamide as its cofactor.

The synthesis of cobamide alone requires 19 enzymes (Supplementary Table 1). It is essential to the acetyl-CoA pathway in acetogens and methanogens. The tetrapyrrole-coordinated Co atom of cobamide in CoFeS accepts a methyl group from an N atom in a pterin cofactor (tetrahydrofolate or tetrahydromethanopterin), and then donates it to a Ni atom in the active site of CODH/ACS.^[191]^ This cobamide-mediated methyl transfer reaction has a Δ*G*°′ of −4 kJ·mol^-1^.^[39]^ Cobamide is also essential for the energy-conserving step of methanogenesis, the Na^+^-pumping membrane complex MtrA-H, which catalyzes the transfer of a methyl group to coenzyme M.^[153]^ In the MtrA-H reaction, the nitrogen-bound methyl group is transferred to CoM in a two-step process involving the corrinoid cofactor. The first step is the transfer of the methyl group from methyl-H_4_MPT to Co(I) (ΔG°′ = −15kJ/mol) and then from methyl-Co(III) to CoM-SH (ΔG°′ = −15kJ/mol) to yield CoM–S–CH_3_ (methyl-CoM). The transfer of the methyl group from methyl-Co(III) is Na^+^-dependent, and is thus implicated in the Na^+^ pumping process.^[192]^ In acetogens^[193]^ that lack cytochromes, net energy conservation from H_2_-dependent CO_2_ reduction is provided by pumping at the reaction catalyzed by Rnf. In methanogens that lack cytochromes, energy conservation occurs at MtrA-H, as previously described.^[190,192,194]^ Figure 4 shows the synthesis of formate, acetate, pyruvate, and methane as products of primordial H_2_-dependent CO_2_ reduction reactions, not the coupled pumping reactions and chemiosmotic energy conservation via the rotor-stator ATP synthase, which are considered later evolutionary inventions. It is likely that primordial energy conservation via the acetyl-CoA pathway entailed acetyl phosphate synthesis and substrate level phosphorylation before the origin of chemiosmotic coupling.^[49,74,195]^

In hydrogenotrophic methanogens there are two routes for the reduction of methenyl-H_4_MPT to methylene-H_4_MPT.^[192]^ The standard route, also used by other methanogens, involves Mtd which uses F_420_H_2_ provided by the activity of Frh, an F_420_-reducing [FeNi] hydrogenase. However, under Ni-limiting conditions, hydrogenotrophic methanogens express an alternative enzyme for methenyl-H_4_MPT reduction: H_2_-forming methylene-H_4_MPT dehydrogenase (Hmd). Hmd reduces methenyl-H_4_MPT with H_2_. Hmd is unique so far in that it is the only H_2_-oxidizing enzyme known that does not reduce FeS clusters or ferredoxin. Instead, it catalyzes the direct transfer of a hydride from H_2_ to methenyl-H_4_MPT with the help of a unique cofactor, the FeGP (iron guanylylpyridinol) cofactor. The biosynthesis of the FeGP cofactor requires an additional seven enzymes,^[196–197]^ which are not included in Figure 4 because it is an alternative pathway. Is the Hmd route ancient, or is it derived? From the standpoint of its constituents, the FeGP cofactor appears to be extremely ancient. The carbon atoms in the FeGP cofactor (excluding the GMP moiety) stem from CO_2_ (including two CO ligands), a methyl group (from S-adenosyl methionine), acetate, and pyruvate. These are all direct products of the acetyl-CoA pathway, including its inorganic precursor^[49]^ as shown in Figure 4, suggesting that the H_2_-oxidizing FeGP cofactor traces to an early stage in (bio)chemical evolution.

The reactions of the acetyl-CoA pathway are replete with carbon metal bonds.^[208]^ The pathway turns up at the center of genomic reconstructions of the last universal common ancestor LUCA.^[141]^ How could such a complex biochemical pathway be genuinely primitive? In 2020 Preiner et al. reported an abiotic analogue of the acetyl-CoA pathway that synthesizes methane, formate, acetate and even pyruvate from H_2_ and CO_2_ without enzymes or organic cofactors. The catalysts were minerals that naturally form in serpentinizing hydrothermal vents,^[209]^ such as awaruite (Ni_3_Fe), magnetite (Fe_3_O_4_), and greigite (Fe_3_S_4_). Ni_3_Fe (and Fe_3_O_4_) generated pyruvate from H_2_ and CO_2_ overnight at 100°C (Figure 4B).^[49]^ Wächtershäuser and others have proposed that single inorganic catalysts might be able to catalyze entire biochemical pathways, for example the reverse TCA cycle or purine synthesis on mineral surfaces.^[43,108]^ In the case of the acetyl-CoA pathway from H_2_ and CO_2_ to pyruvate,^[39,40,74,210]^ we finally have a concrete and reproducible example in hand where it actually happens, the function of 127 proteins (Figure 4) being replaced by a metal that, in nature, is synthesized in serpentinizing hydrothermal vents.^[58]^

The findings of Preiner et al.^[49]^ are not an isolated report. Recent findings by the team of Harun Tüysüz at the Max-Planck Institute for Coal Research in Mülheim have characterized various parameters affecting transition metal-catalyzed H_2_-dependent CO_2_ reduction, as summarized in Table 2. By varying the catalysts, adding silica supports, or altering time and temperature of the reaction, they have been able to obtain high yields of formate and acetate and up to 200 μM pyruvate (Table 2).^[51,111]^

Why is 200 μM pyruvate so important? It is the physiological concentration of pyruvate [180±40 μM] that accumulates in the cytosol of an acetogen, *Clostridium thermoaceticum*, growing on H_2_ and CO_2_.^[204]^ In other words, starting from H_2_ and CO_2_, a solid state transition metal catalyst (Ni) not only produces the most central compound in carbon and energy metabolism, it produces pyruvate at exactly the same concentration as growing cells do. The metal just needs a week^[111]^ (Table 2) instead of 24 hours,^[205]^ the doubling time of *C. thermoaceticum*, to do the job. Do such findings, in sum, identify the origin of autotrophic metabolism from H_2_ and CO_2_ via reactions of the acetyl-CoA pathway?^[40]^ The most direct answer is ‘yes’.

These H_2_-dependent CO_2_ reductions under simulated hydrothermal conditions work without the help of flavin-based electron bifurcation because the redox potential of H_2_ under conditions of serpentinizing vents becomes sufficiently negative (Table 1) to reduce CO_2_ without the need for an additional, supporting oxidant. Although the reactants involved (H_2_ and CO_2_) as well as the catalysts (minerals) are inorganic (Table 2), the organic products are more or less exactly the backbone of carbon and energy metabolism in anaerobic autotrophs (Figure 4). The finding that a geological analogue of the acetyl-CoA pathway, which is the backbone of carbon and energy metabolism of methanogens and acetogens, unfolds spontaneously from H_2_ and CO_2_ activated on transition metal surfaces in hydrothermal conditions, suggests that this pathway is both ancient and the starting point of metabolism. This also indicates that the salient chemical reactions of the acetyl-CoA pathway are older than the enzymes that catalyze them.^[40,210]^

The idea that core metabolic reactions preceded genes in evolution^[33]^ is at the heart of metabolism-first theories for origins and goes back in evidence at least to Degani and Halmann’s 1967 report of non-enzymatic glycolytic reactions,^[211]^ notwithstanding early work on cofactor-catalyzed nonenzymatic reactions from the 1950s.^[74]^ This principle helps to explain the otherwise puzzling observation that most of the enzymes in the archaeal and bacterial versions of methyl synthesis in the acetyl-CoA pathway are not evolutionarily related, even though the chemical reactions and cofactors involved are very similar.^[212]^ The reactions leading to products of the acetyl-CoA pathway (Table 2; Figure 4) start from CO_2_, the product of the Moon-forming impact, and present a strong case for a natural hydrothermal chemistry underlying autotrophic origins, without the need for a late veneer or impactors that generate transiently reducing conditions, because serpentinization, which can also reduce N_2_,^[84]^ has been taking place since there was water on Earth.^[213]^

### 5.3 Rocks and water and CO_2_

Throughout Earth history, H_2_ has always been continuously produced in hydrothermal vents through serpentinization,^[213–214]^ a process in which Fe^2+^-bearing minerals in ultramafic rocks in the Earth’s crust reduce circulating water to molecular hydrogen, generating minerals such as Fe_3_O_4_ and Ni_3_Fe in the process. This process of H_2_ synthesis has been ongoing since the first oceans condensed because the Earth’s ancient crust was largely composed of minerals that can undergo serpentinization^[213]^ and because the process of serpentinization is itself exergonic.^[215–216]^

Starting from pure CO_2_, present in the atmosphere and the oceans as a consequence of the Moon-forming impact, and pure H_2_ from serpentinization, Ni_3_Fe and Fe_3_O_4_ can replace the function of more than 127 enzymes in the synthesis of pyruvate from H_2_ and CO_2_ (Supplementary Table 1). The concept that non-enzymatic versions of chemical reactions at the origin of metabolism were originally catalyzed by minerals,^[33]^ metals^[66,67]^ or alkaline conditions^[211]^ and that they later came to be catalyzed by cofactors and enzymes has been around for over six decades, but the extent to which this very robust principle is realized in core carbon metabolism of microbes continues to be surprising.

Kitadai et al. showed in the laboratory that the production of native metals such as Fe^0^ from the corresponding sulfides can take place under hydrothermal conditions.^[76]^ This is relevant because Varma et al. showed CO_2_ fixation with native metals, such as Fe^0^, Ni^0^ and Co^0^ as catalyst and reductant.^[217]^ A promising prospect involves exploring the chemical capacity of different metal catalysts.^[51,111]^ Another approach relevant to CO_2_ fixation in hydrothermal systems focuses on the electrical currents that are generated between the hydrothermal fluid and seawater, across the mineral deposits of the vents, which act as conductors.^[218–219]^ The discovery of multiple microbial species capable of extracellular electron transfer directly from a solid electron source such as an electrode, rather than a soluble electron-donating molecule,^[220–222]^ opens up questions concerning the potential role for electrotrophic microbial growth on the early Earth. The potentials generated by H_2_ under serpentinizing conditions (Table 1) are themselves a source of electric current, provided that acceptors are available.

## 6 Conclusions

The Moon-forming impact was a key event in Earth’s planetary history. Without it, life on Earth might never have come into existence. Carbon brought to Earth by carbonaceous chondrites was initially present in the form of inert polyaromatic hydrocarbons (PAH).^[223]^ That inert carbon was converted to pure, clean, and reactive atmospheric CO_2_ by the Moon-forming impact. CO_2_ kept the Earth warm enough to maintain liquid surface water, mitigating the faint young Sun, but its main impact was to provide a CO_2_ reservoir that was an accessible carbon source available to kick-start life. When the oceans rained out from the H_2_O-rich atmosphere, CO_2_ started dissolving in the oceans and was destined to subduction as carbonates. The CO_2_ that remained in seawater and that was bound in the crust was available for the first organic syntheses. These required reductant, supplied as H_2_ by serpentinization initiated by water’s first encounters with primordial crust. According to autotrophic theories, synthesis did not occur in a primordial soup. Specific environments and solid state catalysts were required. Today’s submarine hydrothermal vents generate highly localized reducing conditions and allow for the concentration of reactants in far-from-equilibrium environments where H_2_ is continuously formed. Hydrogen gas serves as the reductant for CO_2_, made available by the Moon-forming impact. Transition metal catalysts deposited *in situ* can convert H_2_ and CO_2_ to intermediates and end-products of the acetyl-CoA pathway, such as pyruvate. The acetyl-CoA pathway is assumed to be ancient, likely the first C fixation pathway, and is employed by the oldest microbial lineages – acetogens and methanogens. Theories of autotrophic origins generally aim for congruence between Earth’s early history and geochemistry, on one hand, and microbial physiology, on the other, in order to generate chemical continuity (Morowitz called it ‘historical continuity’^[224]^) in the transition from the first organic chemical reactions to life. Serpentinizing hydrothermal vents link geochemistry with biochemistry in that they combine CO_2_ from the early oxidized atmosphere with a stable source of reductant (H_2_) for organic syntheses in reactions that unfold spontaneously on transition metal surfaces to yield organics that constitute the backbone of carbon and energy metabolism in ancient microbes. They also provide the redox potentials required for CO_2_ reduction without flavin-based electron bifurcation^[58]^ and – were that not enough – they also deposit native metals and native metal alloys *in situ* as inorganic catalysts that specifically accelerate those reactions.^[138,225]^ Life need not have started that way, but had it done so we would be able to recognize the imprint of its origin in reactions of H_2_-dependent chemolithoautotrophic metabolism.

## Figures and Tables

**Table 1:** Some redox potentials for H_2_→2H^+^ + 2e^-^ (values from Suppl. Table 6 in [59]). Note the large effect of pH on *E*. This is because at alkaline pH, the reaction of H^+^ with OH^-^ serves as a pulling reaction, influencing the reaction quotient, and consequently the redox potential.

H_2_ [atm]	pH	Temperature [°C]	*E* [mV]
10	10	100/200	−778/−986
1	10	100/200	−741/−939
0.1	10	100/200	−703/−892
10	9	100/200	−703/−892
1	9	100/200	−666/−845
0.1	9	100/200	−629/−798
10	8	100/200	−629/−798
1	8	100/200	−592/−751
0.1	8	100/200	−555/−704
10	7	100/200	−555/−704
1	7	100/200	− 518/−657
0.1	7	100/200	−481/−610
10	6	100/200	−481/−610
1	6	100/200	−444/−563
0.1	6	100/200	−407/−516
0.01	6	100/200	−370/−469
0.001	6	100/200	−333/−423
0.0001	6	100/200	−296/−376

**Table 2:** Products of aqueous CO_2_ reduction with H_2_ obtained using mineral catalysts.

H_2_:CO_2_ ratio^[a]^	Temp. [°C]	Time [h]	Catalyst^[b]^	Product yields^[c]^			Ref.
				Formate [mM]	Acetate [mM]	Pyruvate [*μ*M]	
4:1^[a]^	100	24	Fe_3_S_4_	2.98	0.43	n.d.^[d]^	[49]
2:3	100	16	Ni_3_Fe	332	0.56	10	[49]
2:3	100	16	Fe_3_O_4_	0.05	0.18	10	[49]
2:3	100	16	Fe_3_O_4_/Fe^0^	1.37	0.27	10	[49]
2:1^[a]^	180	72	Co^[e]^	3.6	1.2	n.d.^[d]^	[51]
2:3	25	24	Ni_3_Fe	26.7	0.04	20	[50]
2:3	100	8	Ni^0^	5.8	0.1	20	[111]
2:3	100	24	Ni_3_Fe	55.5	0.2	40	[111]
2:3	100	24	Ni^0^	36.5	0.7	110	[111]
2:3	100	168	Ni^0^	36.1	0.8	200	[111]

[a] 25 bar in all cases except: In ref. [49] the experiments with greigite were performed at 2 bar; In ref. [51] 2 MPa (20 bar) were used;

[b] In most cases nanoparticular catalysts were used, see references for specifics of catalyst synthesis;

[c] Mean of values reported for the conditions specified;

[d] Not detected;

[e] Cobalt on Ti-modified silica.

## Data Availability

Data sharing is not applicable to this article as no new data were created or analyzed in this study.
